# Craniofacial Reconstruction Evaluation by Geodesic Network

**DOI:** 10.1155/2014/943647

**Published:** 2014-08-20

**Authors:** Junli Zhao, Cuiting Liu, Zhongke Wu, Fuqing Duan, Kang Wang, Taorui Jia, Quansheng Liu

**Affiliations:** ^1^Engineering Research Center of Virtual Reality and Applications, Ministry of Education, Beijing Key Laboratory of Digital Preservation and Virtual Reality for Cultural Heritage, Beijing Normal University, Beijing 100875, China; ^2^College of Software and Technology, Qingdao University, Qingdao 266071, China; ^3^Université de Bretagne-Sud, CNRS UMR 6205, LMBA, Campus de Tohannic, BP 573, 56000 Vannes, France

## Abstract

Craniofacial reconstruction is to estimate an individual's face model from its skull. It has a widespread application in forensic medicine, archeology, medical cosmetic surgery, and so forth. However, little attention is paid to the evaluation of craniofacial reconstruction. This paper proposes an objective method to evaluate globally and locally the reconstructed craniofacial faces based on the geodesic network. Firstly, the geodesic networks of the reconstructed craniofacial face and the original face are built, respectively, by geodesics and isogeodesics, whose intersections are network vertices. Then, the absolute value of the correlation coefficient of the features of all corresponding geodesic network vertices between two models is taken as the holistic similarity, where the weighted average of the shape index values in a neighborhood is defined as the feature of each network vertex. Moreover, the geodesic network vertices of each model are divided into six subareas, that is, forehead, eyes, nose, mouth, cheeks, and chin, and the local similarity is measured for each subarea. Experiments using 100 pairs of reconstructed craniofacial faces and their corresponding original faces show that the evaluation by our method is roughly consistent with the subjective evaluation derived from thirty-five persons in five groups.

## 1. Introduction

Craniofacial reconstruction [1] aims to estimate an individual's face appearance from its skull using the relationship between soft tissues and the underlying bone structure. It has a widespread application in many areas, such as forensic medicine, archeology, medical cosmetic surgery, and public safety. With the development of 3D digitalization technology, the research on computer aided craniofacial reconstruction has widely received attention. The evaluation of the craniofacial reconstruction has an important significance in improving the craniofacial reconstruction methods. However, nearly all researches on craniofacial reconstruction focus on the reconstruction method itself, and little attention is paid to the evaluation method of the reconstruction results.

The craniofacial face is one of the most complex geometric objects in the natural world. How to evaluate the results of the craniofacial reconstruction is still a challenging problem. Existing evaluation methods of the craniofacial reconstruction can be divided into three types: subjective qualitative evaluation method, objective quantitative evaluation method, and the combination method of subjective and objective evaluation. Subjective evaluation methods evaluate the craniofacial reconstruction results subjectively by designing different evaluation strategies. Quatrehomme et al. [2] invited 25 subjects to evaluate and got excellent or good to middle resemblance in 9 out of 25 cases. Snow et al. [3] invited more than 200 respondents to compare a manually recovered craniofacial model with 7 photos; 68% of men and 26% of women gave the correct results. Stephan and Henneberg [4] invited 37 respondents to verify 16 craniofacial reconstruction results; their experimental results also indicated that the recognition rate of the men was higher than that of the women. Helmer et al. [5] invited 24 respondents to compare 24 reconstructed craniofacial faces with their real photos; their results showed that 38% of the reconstruction results were very similar, 17% were similar, 42% were mildly similar, and only one result was considered to be unrelated to the original photo. Although subjective evaluation method is consistent with human cognitive theory, the evaluation process requires a lot of manpower and time, and the accuracy of the evaluation results is influenced by human subjective factors.

Some scholars made a preliminary exploration on evaluating craniofacial reconstruction results by objective method. Feng et al. [6] used a relative angle-context distribution (RACD) to compare the two craniofacial faces. They defined the probability density function of the relative angle-context distribution by counting the number of the relative angles in different intervals. Considering the calculation instability and high time complexity of RACD, Zhu et al. [7] extended the RACD to bending-relative angle-context distribution (BRACD) algorithm by measuring the bending of a reference hemisphere to a craniofacial model. Duan et al. [8, 9] analyzed the correlation between the skull and face shape and measured the craniofacial reconstruction error by the distance of corresponding points between the reconstructed craniofacial face and the original face.

Several researchers combined subjective and objective evaluations. For example, VaneZis [10] invited 20 assessors to choose the three best matches from 10 reconstructed craniofacial faces of one skull with the original face. They also computed the correlation between the subjective results with the objective evaluation results by mathematical shape analysis assessment using Procrustes Analysis. The results are not statistically significant but indicate that the objective method does seem to capture some perceptual similarity in human observers. Moorthy et al. [11] also proposed a combination evaluation method. In subjective aspect, they conducted a subjective study, where a set of human subjects (12 subjects on 180 3D faces) rated the similarity of pairs of faces (a total of 5490 pairs of similarity scores). In objective aspect, they extracted Gabor features from automatically detected feature points on the range and texture images from 3D faces. Finally, they demonstrated that these features correlated well with human judgment of similarity.

In this paper, we propose a new global and local evaluation method of craniofacial reconstruction based on the geodesic network. We define the weighted average of the shape index value in a neighborhood as the feature of one vertex. The absolute value of the correlation coefficient of the feature of all corresponding geodesic network vertices between two models is taken as the similarity. It lays a foundation for qualitative and quantitative analysis of craniofacial reconstruction results and provides guidance for improvement of the craniofacial reconstruction methods.

The rest of this paper is organized as follows. The materials and methods are presented in Section 2.  Section 3 presents experimental results. Some conclusions are provided in Section 4.

## 2. Materials and Methods

### 2.1. Materials

**Figure 1:**
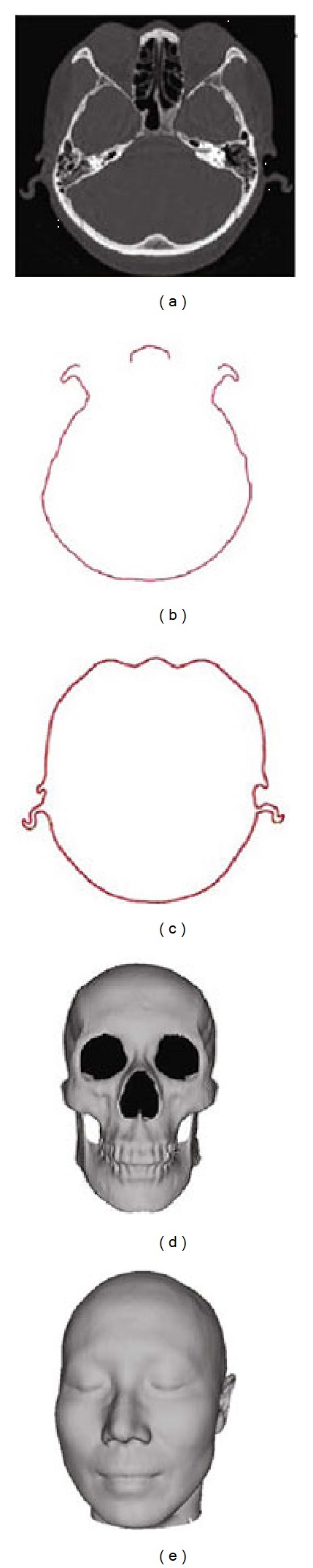
Data acquisition. (a) The head slice captured by CT scanner. ((b)-(c)) The extracted skull and skin contour. ((d)-(e)) The reconstructed 3D skull and face triangle mesh models.

**Figure 2:**
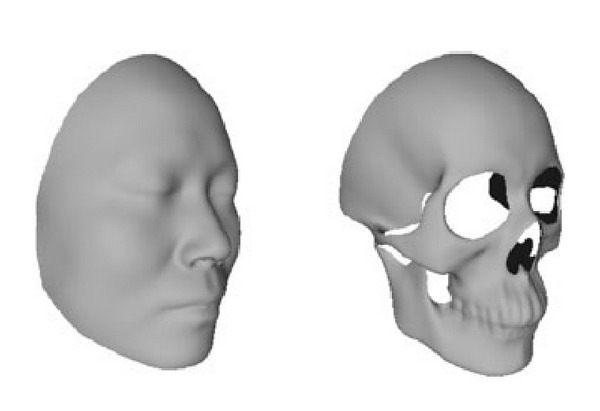
The reference skull and face skin for registration.

This study was approved by the Institutional Review Board (IRB) of Image Center for Brain Research, National Key Laboratory of Cognitive Neuroscience and Learning, Beijing Normal University. The study was carried out on a database of 208 whole head CT scans on voluntary persons that mostly come from Han ethnic group in North of China, aged from 19 to 75 years. The CT scans were obtained by a clinical multislice CT scanner system (Siemens Sensation16) in Xianyang Hospital located in western China. As Figure 1 shows, we firstly extract skull and face borders from the original CT slice images and then reconstruct the triangle mesh models of the 3D skull and skin surfaces by a marching cubes algorithm [12]. More details on the procedure of the data processing can be found in [13]. The obtained three-dimensional craniofacial mesh models often contain defects such as holes, gaps, degeneracies, or nonmanifold configurations. We need to fill holes and gaps and remove the scattered points to make the 3D face model become a full and well-structured manifold. To eliminate the effects of data acquisition, posture, and scale, all 3D craniofacial data are transformed into a unified Frankfurt coordinate system [14]. Finally, as Figure 2 shows, we select a craniofacial data as a reference template and cut away the back part of the reference craniofacial model considering that there are too many vertices in the whole head and the face features mainly concentrate on the front part of the head. All of the craniofacial models are registered with the reference model automatically by the nonrigid data registration method in [14].

### 2.2. Overview of the Method

The craniofacial reconstruction evaluation is to assess the similarity between the reconstructed craniofacial face and the original face. We propose an objective method of the craniofacial reconstruction evaluation based on the shape index of the geodesic network vertices. The method evaluates the similarity both globally and locally. The geodesic network of the reconstructed craniofacial face and the original face are built, respectively, by geodesics and isogeodesics, whose intersections are geodesic network vertices. The weighted average of the shape index values within a neighborhood of each geodesic network vertex is computed for each craniofacial face model. The weighted average is considered as the feature of the vertex. The absolute value of the correlation coefficient of the features of all corresponding geodesic network vertices is taken as the holistic similarity between two models. To evaluate the reconstruction locally, these geodesic network vertices of each model are divided into six subareas, forehead, eyes, nose, mouth, cheeks, and chin. The absolute value of the correlation coefficient of the features of corresponding network vertices in each subarea between two craniofacial models is taken as the local similarity.

### 2.3. Geodesic Network Construction

Geodesic is the curve with geodesic curvatures equal to zero. It is the shortest path between two points on a surface. Due to its intrinsic invariance, geodesic can be applied to face recognition that is insensitive to expression. According to geodesic, a geodesic polar coordinate system can be constructed by exponential mapping [15, 16]. Inspired by this idea, we construct the geodesic network according to geodesics and isogeodesics, respectively, on two craniofacial facial models and take the intersection points of geodesics and isogeodesics, that is, geodesic network vertices, as the corresponding points between the two craniofacial face models. Thus, we can compare the similarity of the two models through the features of the corresponding geodesic network vertices.

The detailed steps for constructing the geodesic network are described as follows. Firstly, we find the nose tip of the craniofacial face model and make it as the source point of geodesics. Because the craniofacial models are frontal, the nose tip point **O** is the highest point of the whole model, that is, the point which has the biggest *y* value (or *z* value) in Frankfurt coordinate. Secondly, *n* isogeodesics are extracted according to the geodesic distance to the source point. The extracted *n* isogeodesics are well-distributed with the same interval between two of them. The interval is one out of *n* of the shortest geodesic distance from the nose tip point to the boundary point set *B*. So the isogeodesics set IG can be obtained by the following formula:

(1) $$\begin{array}{c} \text{IG}=\left\{{\text{IG}}_{j}\;|\;{\text{IG}}_{j}=\text{isogeodesic}\left(O,d_{X}^{j}\right)\right\},\\ d_{X}^{j}=j\times \left(\frac{\min_{B_{i}\in B}\;d_{X}\left(O,B_{i}\right)}{n}\right),\;j=1,2,\ldots ,n, \end{array}$$

where isogeodesic  (**O**, *d*_*X*_^*j*^) represents the isogeodesic that the geodesic distance from the source point **O** to the point on the curve is equal to *d*_*X*_^*j*^. Geodesic distance can be solved by the existing geodesic algorithms, such as MMP [17], ICH [18], PCH [19], and SVG [20]. Here, we adopt the classical MMP algorithm, which has been realized and described in detail in [21]. Thirdly, *m* geodesics are computed from the tip nose according to an equal angular interval. The equal angle can be found as follows: (1) the direction of the nose tip and the middle point of eyebrows is selected as the initial direction; (2) the outermost isogeodesic is projected into the tangent plane of the nose tip, and the cross points of the equal angular line from the nose tip with the projection of the outermost isogeodesic on the tangent plane are computed; (3) project the cross point into original outermost isogeodesic, and these equal division points **P**_*i*_ are taken as the target points of geodesics; (4) geodesic *G*_*i*_ from the nose tip to the target points **P**_*i*_ can be computed as follows:

(2) $$\begin{array}{c} G=\left\{G_{i}\;|\;G_{i}=\text{geodesic}\left(O,P_{i}\right),\left(i=1,2,\ldots ,m\right)\right\}. \end{array}$$

Finally, the intersection points *Q*_*ij*_ of the geodesics *G*_*i*_ and isogeodesics IG_*j*_ are calculated, and all intersection points constitute the intersection point set *Q*, that is, geodesic network point set:

(3) Q={Qij ∣ Qij∈Gi∩IGj},(i=1,2,…,m;j=1,2,…,n).

**Figure 3:**
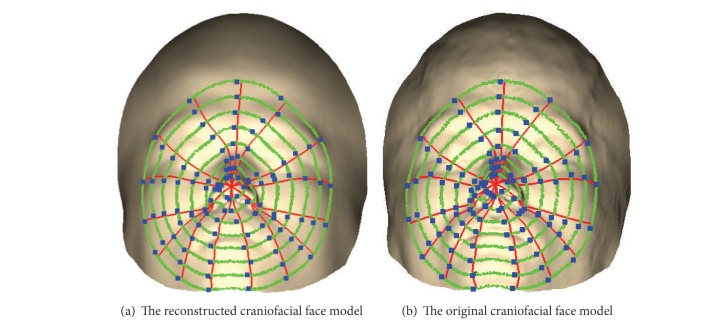
Geodesic network vertices of the reconstructed and the original craniofacial face models.

Figure 3 shows the geodesic networks of the reconstructed and the original craniofacial face models. Let *Q*_1_ and *Q*_2_ be the geodesic network point sets of the craniofacial models *M*_1_ and *M*_2_, respectively. The geodesic network point *Q*_*ij*_^1^ of *M*_1_ and *Q*_*ij*_^2^ of *M*_2_ are the corresponding points of the two craniofacial face models since they have the same initial directions and the same source (the nose tip).

### 2.4. Extracting Shape Index Features

Due to the intrinsic invariance of geodesic, the features of the geodesic network vertices can be used to evaluate the results of craniofacial reconstruction. Shape index (*S*_*I*_) is the feature generated by the principal curvatures *κ*_1_ and *κ*_2_. It can capture the “local” shape of a surface. Thus, we evaluate the craniofacial reconstruction based on the correlation coefficient of the shape index value of the corresponding geodesic network vertices between the original and reconstructed craniofacial faces. Shape index *S*_*I*_ is defined by the curvedness-orientation-shape map on sphere (COSMOS) representation [22] and can quantitatively measure the shape of a surface at a point **P**. Every distinct surface shape corresponds to a unique value of *S*_*I*_. At a point **P**, the shape index *S*_*I*_ is defined as

(4) $$\begin{array}{c} S_{I}\left(P\right)=\frac{1}{2}-\frac{1}{\pi }\text{ta}{\text{n}}^{-1}\left(\frac{\kappa_{1}\left(P\right)+\kappa_{2}\left(P\right)}{\kappa_{1}\left(P\right)-\kappa_{2}\left(P\right)}\right). \end{array}$$

For the corresponding network vertexes **P** and **P**′ at the original and the reconstructed craniofacial models, the shape index values are *SO*(**P**) and *SR*(**P**′), respectively. To avoid the interference of the noise and obtain the robust shape index value, we define the weighted average of the shape index value in a neighborhood as the feature of one vertex. Owing to the important role of the geodesic network vertex in evaluating the two craniofacial models, the weight of the geodesic network vertex itself should be the highest, and the weights of the 1-ring neighborhood of the vertex should be higher than those of the 2-ring neighborhood. In this paper, we choose the weights of 100, 10, and 1 for the network vertex, 1-ring neighborhood, and 2-ring neighborhood, respectively. The features of the corresponding network vertex **P** and **P**′ of the two craniofacial models are $SO(\bar{P})$ and $SR(\bar{P^{\prime }})$, respectively. For the two whole models, the features of all geodesic vertices constitute, respectively, the vectors **S** **O** = (*SO*_1_, *SO*_2_,…, *SO*_*N*_) and **S** **R** = (*SR*_1_, *SR*_2_,…, *SR*_*N*_) where *N* is equal to *n* × *m*, that is, the product of the number of isogeodesics and the number of geodesics.

### 2.5. Evaluating the Craniofacial Reconstruction Globally and Locally

Generally, the correlation coefficient is used to measure how two sets of variables are linearly related. Here, we use the correlation coefficient to measure the similarity of two craniofacial models. For evaluating the craniofacial reconstruction result globally, we compute the correlation coefficient of the features of all corresponding geodesic network vertices between the original and the reconstructed craniofacial models by the following formula:

(5) $$\begin{array}{c} R\left(SO,SR\right)=\frac{\sum_{i=1}^{N}\left(SO_{i}-\bar{SO}\right)\left(SR_{i}-\bar{SR}\right)}{\sqrt{\sum_{i=1}^{N}{\left(SO_{i}-\bar{SO}\right)}^{2}\sum_{i=1}^{n}{\left(SR_{i}-\bar{SR}\right)}^{2}}}. \end{array}$$

The value of correlation coefficient ranges in the interval [−1,1]. We take the absolute value as the similarity measure. The smaller the value is, the weaker the similarity is.

**Figure 4:**
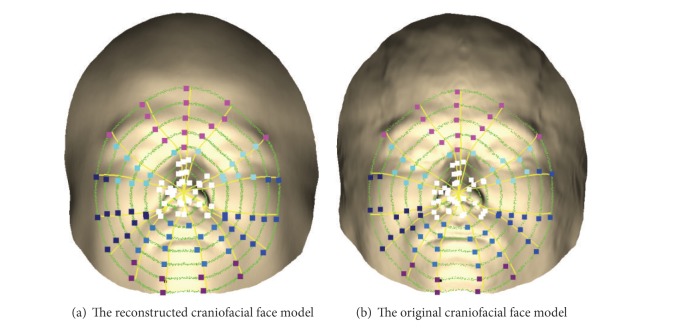
Six parts of the geodesic network vertices: forehead area is in pink color, eyes area is in cyan color, nose area is in white color, cheek area is in dark blue color, mouth area is in blue color, and the chin area is in wine color.

The global evaluation reflects only the whole similarity between the reconstructed and the original craniofacial models and cannot discern whether the region is well reconstructed or not. In order to evaluate the reconstructed craniofacial model locally, we divide the geodesic network vertices into six parts, that is, forehead, eyes, nose, mouth, cheeks, and chin (as shown in Figure 4). The features of the geodesic network vertices in each subarea are used to compute the similarity measure of the subarea by (5). According to the local evaluation, we can make a statistical analysis of the local similarity measure and give some feedback to promote the craniofacial reconstruction methods.

## 3. Experiments and Discussion

To acquire the experimental data, we use the partial least squares regression (PLSR) method [8] to reconstruct the craniofacial models, where 108 pairs of skulls and face skins among the 208 CT scans are used as the training data, and the other 100 skulls are used as the test data for the craniofacial reconstruction. Thus, we obtain 100 pairs of the reconstructed face models and the corresponding original craniofacial models. To compare with the subjective evaluation, we first introduce the subjective evaluation procedure we designed. Then, the reconstruction results are evaluated globally and locally by the proposed objective method.

### 3.1. Subjective Evaluation

**Figure 5:**
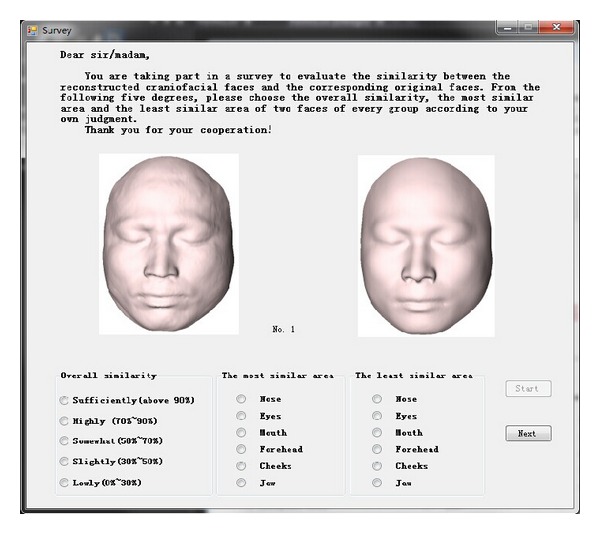
Survey.

In order to evaluate the proposed objective method, we invited 35 subjects to evaluate the 100 reconstructed craniofacial face models. 100 pairs of craniofacial face models were divided into five groups and each group had 20 pairs. 35 subjects were divided into five groups, and each group had seven subjects to evaluate twenty pairs of reconstructed craniofacial faces and corresponding original craniofacial faces. Each subject was asked to observe every pair of faces on the screen and choose the overall similarity degree from the following five degrees as Figure 5 shows: sufficiently (above 90%), highly (70%~90%), somewhat (50%~70%), slightly (30%~50%), and lowly (0%~30%). They were also asked to select the most similar area and the least similar area from the following six subareas: nose, eyes, mouth, forehead, cheeks, and chin. To avoid visual fatigue, each subject was only in charge of evaluating twenty pairs of craniofacial faces. According to the subjective assessing from the five groups, the similarities of 100 pairs of craniofacial faces were gained, and each pair had seven similarity degrees by seven different subjects. We computed the mean maximum similarity score according to the upper limits of the seven similarity degrees and the mean minimum similarity score by the lower limits. Thus, we obtained a similarity interval for each pair of craniofacial faces by the subjective evaluation. In the following, if the similarity obtained by the objective method is in the interval for one pair of craniofacial faces, we think the evaluation is consistent with the subjective evaluation.

### 3.2. Global Evaluation

The global evaluation is to compare the features of all geodesic network vertices to get the objective similarity score between the reconstructed craniofacial face and the corresponding original face.  Table 1 shows the similarity scores by the objective and subjective assessments. From Table 1, we can see that the similarity scores by the objective assessment are within the similarity interval of the subjective evaluation. The assessment results show that the objective assessment is consistent with the human subjective evaluation.

### 3.3. Local Evaluation

Local evaluation is the evaluation of the similarity in six subareas: forehead, nose, eyes, mouth, cheeks, and chin between the reconstructed craniofacial face and the corresponding original craniofacial face. We find the most and the least similar areas and compare them with the subjective assessments. We take three pairs of craniofacial faces in Table 1; the local similarity scores are computed by the features of the geodesic network vertices in each subarea.  Table 2 shows the evaluation results by the objective method. We can see that the nose area is the most similar area and the eyes area is the least similar area for the case 0401; the mouth area is the most similar area and the forehead area is the least similar area for the case 001-2354; the cheeks area is the most similar area and the mouth area is the least similar area for the case 3718. These results are consistent with the subjective evaluation.

**Figure 6:**
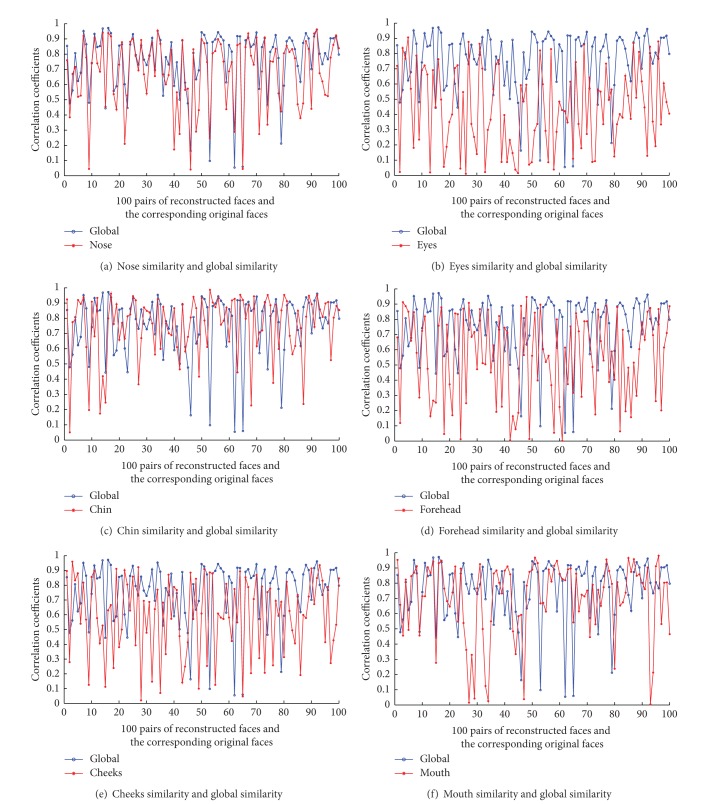
The similarity comparisons of nose, eyes, mouth, forehead, cheeks, and chin with the global similarity of 100 pairs of the reconstructed craniofacial faces and the original craniofacial faces by objective method.

We evaluate all of the 100 reconstructed craniofacial faces locally and compare the local evaluation of six subareas with the global evaluation.  Figure 6 shows the comparisons. From Figure 6, we can see that the nose similarity is roughly consistent with the global similarity and the similarity of eyes is almost unrelated with the global similarity. We also compute the absolute value of correlation coefficients between the local similarities of the six subareas with the global similarities using the l00 cases.  Table 3 shows the absolute value of correlation coefficients. From Table 3, we can also see that the similarity of the nose area is highly interrelated with the global similarity and the eyes area is the region of the lowest correlation with the global similarity. Compared with other subareas, the nose area has more of the geodesic network vertices and the curvature of the nose area changes more significantly; so the similarity of nose is closer to the global similarity. The eyes area has fewer geodesic network vertices; thus the similarity of eyes has a little correlation with the global similarity.

We compute the mean similarity score of each subarea of the 100 pairs of faces. The mean similarity scores are shown in Table 4. We can see that the maximum is in the chin area and the minimum is in the eyes area. This indicates that the eyes area is not well reconstructed. These objective evaluation results are consistent with the subjective evaluation results.

## 4. Conclusions

Craniofacial reconstruction has a widespread application in forensic medicine, archeology, medical cosmetic surgery, and so forth. However, nearly all researches on craniofacial reconstruction focus on the reconstruction method itself, and little attention is paid to the evaluation methods of the reconstruction. This paper proposes an objective method to evaluate globally and locally the reconstructed craniofacial faces based on the shape index of geodesic network vertices. The geodesic networks of the craniofacial faces are built by geodesics and isogeodesics. For each geodesic network vertex, we define the weighted average of the shape index value in a neighborhood as the feature of the network vertex. The absolute value of the correlation coefficient of the feature of all corresponding geodesic network vertices between two models is taken as the similarity. We used 100 pairs of reconstructed craniofacial faces and their corresponding original faces to evaluate our method. To compare with the subjective evaluation, we also invited 35 subjects to evaluate visually the reconstructed craniofacial faces. Experimental results show that the evaluation by our method is roughly consistent with the subjective evaluation. By evaluating the craniofacial reconstruction effects both globally and locally, we can provide guidance for improvement of the craniofacial reconstruction methods. In addition, since small face expression can be regarded as the isometric deformation, under which the geodesic distance is invariant, the proposed method is also fit for the craniofacial faces of small expression variation.

## Figures and Tables

**Table 1:** The global similarity comparison results between restored craniofacial faces and original faces.

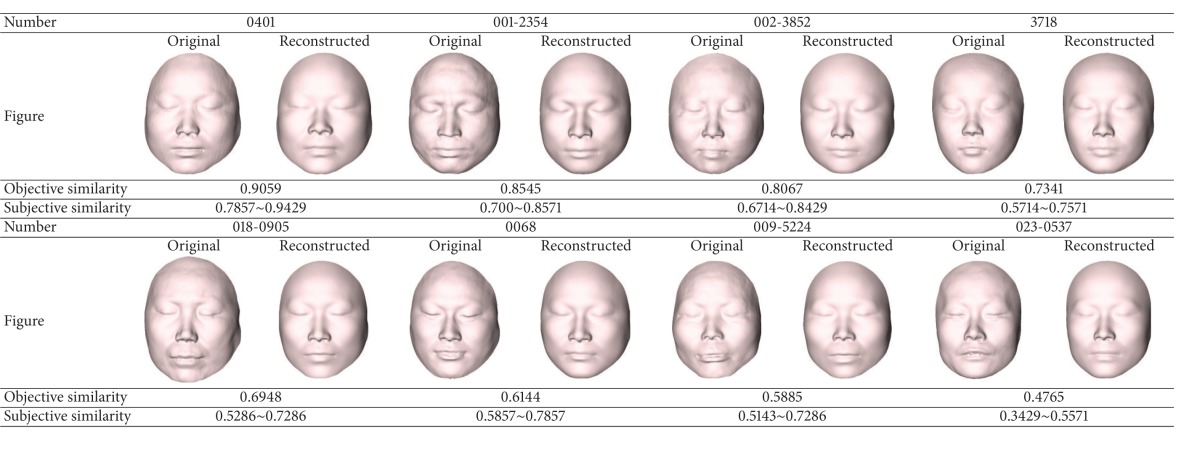

**Table 2:** The local similarity comparison results between restored craniofacial faces and original faces.

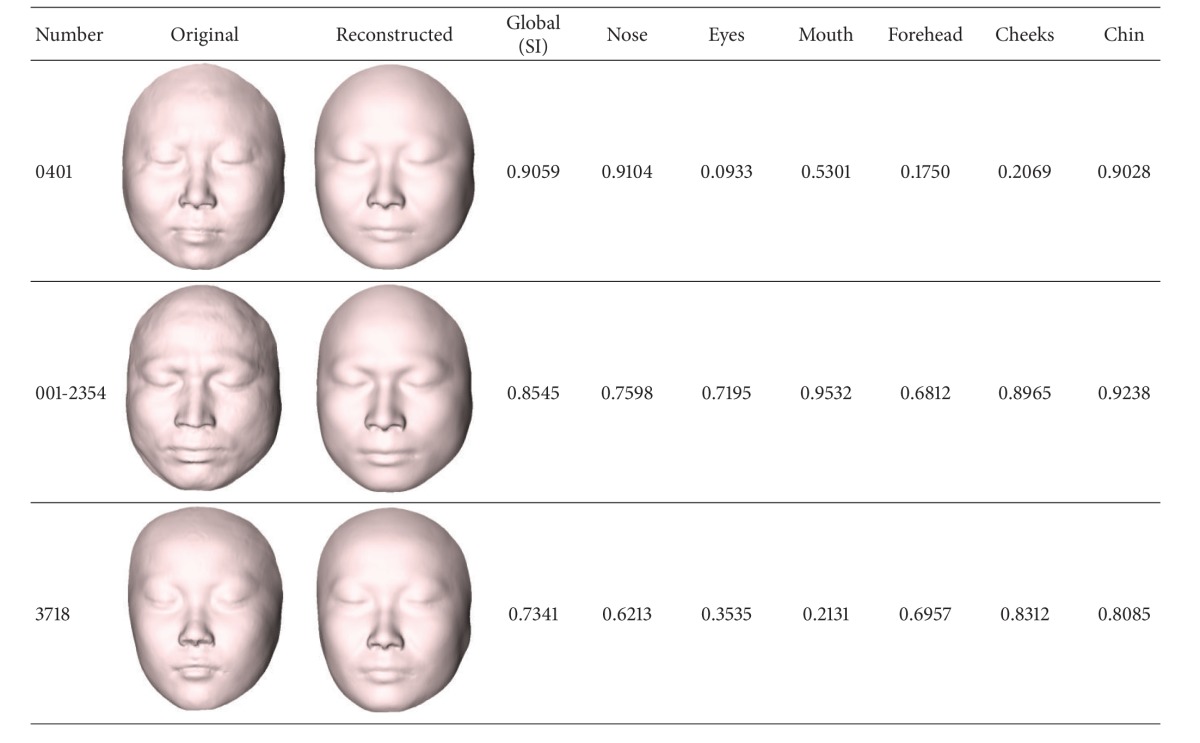

**Table 3:** The correlation coefficients between forehead, nose, eyes, mouth, cheeks, and chin similarity with the global similarity of 100 pairs of reconstructed craniofacial faces and the corresponding original faces.

*R*	Nose	Mouth	Cheeks	Chin	Forehead	Eyes
Global	0.9150	0.1759	0.0683	0.0317	0.0305	0.0055

**Table 4:** The mean similarity score of each subarea of the 100 pairs of faces.

Area	Global	Nose	Mouth	Cheeks	Chin	Forehead	Eyes
Mean similarity	0.7565	0.6763	0.6993	0.5925	0.7513	0.5476	0.4400
